# Regarding ‘experiences of young adults affected by cancer within an 8-week yoga intervention delivered by videoconference: a qualitative interview study’

**DOI:** 10.1080/07853890.2024.2443556

**Published:** 2024-12-23

**Authors:** Heng Zhou, Yilin Jiang, Zhanshuo Xiao

**Affiliations:** a Department of Student Affairs, Chongqing Medical University, Yuzhong, Chongqing, China; b School of Education, Universiti Teknologi Malaysia, Skudai, Johor, Malaysia; c Department of Dermatology, Guanganmen Hospital, China Academy of Chinese Medical Sciences, Beijing, China

Dear editors

We meticulously reviewed the study ‘Experiences of young adults affected by cancer within an 8-week yoga intervention delivered by videoconference: a qualitative interview study’ [1]. This article provides an important contribution to the field of oncology care by highlighting the potential of yoga as a supportive intervention for young adults. This demographic often faces distinct physical, psychological and emotional challenges during and after cancer treatment, which differ significantly from older adults. By demonstrating the role of yoga in enhancing both mental and physical well-being through self-care, mindfulness and peer connection, the study underscores the necessity of age-appropriate and accessible interventions tailored to the unique needs of young cancer survivors.

However, despite the strengths of the study, there are several notable limitations that warrant attention. First, the exclusive reliance on a videoconference delivery model, while a practical solution during the COVID-19 pandemic, may have restricted the depth of embodied engagement – a core aspect of yoga’s therapeutic effect. Yoga practice traditionally emphasizes physical presence, non-verbal communication, and interpersonal connection, which could be compromised by the digital format [2, 3]. While the convenience of remote participation is valuable, the lack of in-person interaction may have limited the participants’ ability to fully experience the mind–body integration central to yoga. Future interventions could explore hybrid models or advanced digital solutions such as augmented reality to more closely replicate the immersive, in-person experience while retaining the accessibility of online formats.

Second, the study’s recruitment strategy raises concerns about representativeness. With recruitment primarily through social media and patient advocacy networks, there is a risk of excluding participants from marginalized backgrounds, particularly those with limited access to digital technologies or from lower socioeconomic strata [4]. This could result in an underrepresentation of voices from more disadvantaged groups, potentially limiting the generalizability of the findings. Collaborating with healthcare providers and community organizations, as well as employing more diverse recruitment strategies, could help to ensure a broader, more inclusive participant base in future studies.

Moreover, the relatively short duration of the intervention – eight weeks – may not be sufficient to capture the long-term benefits or challenges associated with yoga practice. Cancer survivors often face prolonged physical and psychological difficulties well beyond their immediate treatment, such as chronic fatigue, pain, or psychological distress [5]. The study provides valuable insights into short-term improvements, yet it remains unclear how sustained yoga practice might affect longer-term outcomes, such as recurrence anxiety or chronic symptom management. Longitudinal studies that extend the observation period beyond eight weeks are needed to assess the durability of yoga’s benefits over time, as well as its potential role in long-term recovery and quality of life.

In conclusion, despite these limitations, this study represents a significant advancement in understanding the supportive role of yoga for young adults with cancer. The findings provide a solid foundation for the continued development of holistic and accessible care interventions that can meaningfully improve the quality of life for this vulnerable population. The authors should be commended for their work, which opens valuable pathways for future research and clinical application in oncology care.

## Data Availability

Data sharing is not applicable to this article as no new data were created or analyzed in this study
